# Influence of *COMT* genotype and affective distractors on the processing of self-generated thought

**DOI:** 10.1093/scan/nsu118

**Published:** 2014-09-03

**Authors:** Emma J. Kilford, Iroise Dumontheil, Nicholas W. Wood, Sarah-Jayne Blakemore

**Affiliations:** ^1^Institute of Cognitive Neuroscience, University College London, 17 Queen Square, London, WC1N 3AR, UK, ^2^Department of Psychological Sciences, Birkbeck, University of London, Malet Street, London, WC1E 7HX, UK and ^3^Department of Molecular Neuroscience, Institute of Neurology, University College London, Queen Square, London, WC1N 3BG, UK

**Keywords:** COMT, executive function, affective processing, behavioural genetics, prefrontal cortex

## Abstract

The catechol-O-methyltransferase (COMT) enzyme is a major determinant of prefrontal dopamine levels. The Val^158^Met polymorphism affects COMT enzymatic activity and has been associated with variation in executive function and affective processing. This study investigated the effect of *COMT* genotype on the flexible modulation of the balance between processing self-generated and processing stimulus-oriented information, in the presence or absence of affective distractors. Analyses included 124 healthy adult participants, who were also assessed on standard working memory (WM) tasks. Relative to Val carriers, Met homozygotes made fewer errors when selecting and manipulating self-generated thoughts. This effect was partly accounted for by an association between *COMT* genotype and visuospatial WM performance. We also observed a complex interaction between the influence of affective distractors, *COMT* genotype and sex on task accuracy: male, but not female, participants showed a sensitivity to the affective distractors that was dependent on *COMT* genotype. This was not accounted for by WM performance. This study provides novel evidence of the role of dopaminergic genetic variation on the ability to select and manipulate self-generated thoughts. The results also suggest sexually dimorphic effects of *COMT* genotype on the influence of affective distractors on executive function.

A wide variety of cognitive processes are associated with the prefrontal cortex (PFC), including social cognition, executive function, working memory (WM) and emotional regulation (Williams and Goldman-Rakic, 1995; Ochsner and Gross, 2005; Frith and Frith, 2008; Cools and D’Esposito, 2011). These processes and the neural systems associated with them are impaired in many psychiatric disorders (Millan *et al.*, 2012). Converging evidence suggests that executive function and WM are strongly influenced by the dopaminergic system, with cognitive performance being related to prefrontal dopamine levels (Goldman-Rakic, 1998; Cools and D’Esposito, 2011). However, less is known about the relationship between prefrontal dopamine and socio-affective cognition (Skuse, 2006).

Dopamine levels in the brain are regulated by enzymes that degrade dopamine and other catecholamines. The catechol-O-methyltransferase (COMT) enzyme is a major determinant of dopamine function in the PFC, due to low expression of other regulatory enzymes in this brain region (Meyer-Lindenberg and Weinberger, 2006; Tunbridge *et al.*, 2006). Functional genetic polymorphisms can alter enzyme activity levels, by affecting the rate of transcription of the protein or its amino-acids sequence. Rs4680, the most commonly studied single nucleotide polymorphism of the *COMT* gene, consists of a substitution of Valine with Methionine at codon 158 of the *COMT* gene (Val^158^Met; Lachman *et al.*, 1996) and results in a 40% reduction of COMT enzymatic activity in Met homozygotes compared with individuals who are homozygous for the ancestral Val allele (Chen *et al.*, 2004).

The lower COMT activity of Met homozygotes has been associated with greater levels of prefrontal extracellular dopamine, and also with superior performance on tasks assessing executive function and WM, and reduced PFC activation during such tasks, compared with carriers of the Val allele (see Tunbridge *et al.*, 2006; Dickinson and Elvevåg, 2009; Mier *et al.*, 2010; Witte and Flöel, 2012 for reviews). Conversely, it has been proposed that the Val allele confers an advantage for emotional processing and regulation (Goldman *et al.*, 2005), with Met carriers showing increased reactivity to aversive stimuli (Mier *et al.*, 2010) and potentially being at an elevated risk for emotion-related psychopathology (see Montag *et al.*, 2012 for review).

Relationships between *COMT* genotype and behaviour have not always been replicated (Barnett *et al.*, 2008; Dickinson and Elvevåg, 2009; Montag *et al.*, 2012; Witte and Flöel, 2012). Possibly contributing to this variation are the sexually dimorphic effects of *COMT* genotype, which have been observed on both neurochemical and behavioural measures (Gogos *et al.*, 1998), and on associations with psychopathology (Harrison and Tunbridge, 2008). For example, the Met allele is associated with obsessive-compulsive disorder in males but not females (Pooley *et al.*, 2007). The basis of the sexual dimorphism of COMT effects is not well understood, but may in part be explained by the regulation of *COMT* expression by oestrogen, and sex differences in baseline dopamine levels, among other mechanisms (Gogos *et al.*, 1998; Harrison and Tunbridge, 2008).

The current study focused on individual differences in an aspect of executive function that has not previously been investigated in relation to *COMT* genotype: the flexible modulation of the balance between the processing of self-generated information and cognitive processes provoked by perceptual experience. This includes the ability to resist distraction from perceptual stimuli when processing self-generated information (i.e. information occurring in the absence of sensory input), and the ability to select, maintain and manipulate self-generated thoughts (Burgess *et al.*, 2007). These abilities can be studied using the Alphabet Task, a paradigm in which participants process perceptually derived and self-generated information in alternating blocks (Gilbert *et al.*, 2005).

The modulation of attention towards or away from emotionally salient information has been implicated in emotion-related psychopathology, although there are different theories regarding the precise nature of such effects (Bar-Haim *et al.*, 2007; Ladouceur *et al.*, 2009). Establishing the role of dopamine in the interplay between executive function and socio-affective processing will contribute to a better understanding of the mechanisms by which genetic variants may confer risk for poor emotional regulation and affective disorders. Therefore, a second aspect of the present study was the incorporation of socio-affective distractors to investigate the hypothesis that *COMT* genotype is associated with differential sensitivities to emotionally salient material, and to assess the influence of socio-affective distractors on the selection and manipulation of self-generated information compared with perceptually derived information.

We hypothesized that, due to the relationship between prefrontal dopamine and executive function, individuals homozygous for the Met allele would show specific superior processing of self-generated information on the Alphabet Task, accounted for by increased PFC dopamine availability. Following studies suggesting the Met allele may confer increased risk for emotion-related psychopathology, it was also hypothesized that effects of socio-affective distractors on Alphabet task performance would be further moderated by *COMT* genotype, with Met homozygotes being more likely to be affected by negative distractors.

## METHODS

### Participants and genetic analysis

We recruited 161 adults (81 male) from UCL volunteer databases, all of whom were healthy according to self-report. The study was approved by the UCL Research Ethics Committee, and all participants gave written informed consent. Participants were tested individually on behavioural tasks and subsequently provided a saliva sample, which was genotyped for the rs4680 Val^158^Met substitution on the *COMT* gene (see Supplementary Materials for details of the genetic analysis). Effects of *COMT* genotype were explored using a Val dominant model (Met/Met *vs* Val carriers). This genotype model was chosen based on previous research findings that this model was the most effective in explaining variance in behaviour (Barnett *et al.*, 2007; Dumontheil *et al.*, 2011).

To increase the homogeneity of the sample, East Asian participants were excluded from all analyses, as the Val allele is significantly more frequent in East Asian populations than European, African and Southwest Asian populations (Palmatier *et al.*, 1999). After exclusions based on ethnicity, failed genotyping (see Supplementary Materials for further details) and poor task performance (see ‘Statistical Analyses’ section for exclusion criteria) analyses included 124 participants (Table 1). There were no significant differences in self-reported state or trait anxiety (Spielberger *et al.*, 1983), verbal IQ (Wechler, 1999), sex or ethnicity between the genotype groups (*P* values > 0.156); however, age differed significantly (*t*(122) = 2.01, *P* = 0.047), with Met homozygotes being younger by 1.5 years than Val carriers on average. Therefore, analyses were repeated with age included as a covariate to assess whether age differences accounted for any genotype effects (Table 1; further details of group matching can be found in the Supplementary Materials).

**Table 1:** Participant demographics

*COMT* genotype		Age	Verbal IQ	Self-reported anxiety		Sex		Ethnicity	
				State	Trait	Female	Male	Caucasian	Non-Caucasian
	*n*	*M* (s.d.)	*M* (s.d.)	*M* (s.d.)	*M* (s.d.)	*n*	*n*	*n*	*n*
Met/Met	36	25.2 (3.2)	116.7 (14.4)	35.6 (8.5)	43.7 (11.5)	15	21	28	8
Val carriers	88	26.7 (3.9)	115.7 (14.2)	35.1 (10.4)	41.5 (10.9)	49	39	57	31
Total	124	26.3 (3.8)	116.0 (14.2)	35.3 (9.9)	42.1 (11.1)	64	60	85	39

Mean age, verbal IQ, self-report anxiety and distribution of sex and ethnicity are presented for each *COMT* genotype group and the whole sample.

### Behavioural tasks

Participants performed the Emotional Alphabet task in the second position out of a set of five cognitive tasks. Two tasks were standard measures of WM, a visuospatial WM (VSWM) grid task and a Backwards Digit span task, and are described in detail in Dumontheil *et al.* (2014). Performance on these tasks is presented here only in relation to performance on the Emotional Alphabet task. The other two tasks, which focused on social cognitive processes, along with two additional questionnaire measures, are described elsewhere (Dumontheil *et al.*, 2014). Verbal IQ was assessed using the vocabulary subtest of the Wechsler’s Abbreviated Scale of Intelligence (Wechsler, 1999). Trait and state anxiety were assessed with the State-Trait Anxiety Inventory for Adults self-report questionnaire (Spielberger *et al.*, 1983). The entire testing session lasted approximately 1 h.

#### Emotional Alphabet task

**Fig. 1:**
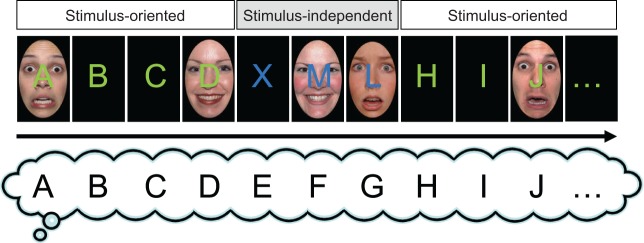
Emotional Alphabet task. In stimulus-oriented (SO) blocks, participants made ‘yes’/‘no’ judgements about the shape of green letters presented on the screen in alphabetical order. In stimulus-independent (SI) blocks, participants had to ignore the blue letters on the screen, continue the alphabet sequence in their head (e.g. ‘E’, ‘F’, ‘G’, bottom row) and make the judgement about the letter in their head. In half of all trials, an emotional distractor was present; either a fearful face or a happy face was presented behind the letter.

This task was adapted from the Alphabet Task (Gilbert *et al.*, 2005), which tests the control of the allocation of attention between perceptually derived (stimulus-oriented, SO) and self-generated (stimulus-independent, SI) information. SO phases of the task require participants to attend to and process information presented on a computer screen, while SI phases require participants to ignore this information and instead attend to and process self-generated information. The adapted task had a factorial design, with two within-subjects factors: block type (SO, SI) and distractor type (no distractor, fearful faces, happy faces; Figure 1).

In SO blocks, participants performed a shape judgement about a green letter presented on the screen. After each response, a new letter was presented, following the sequence of the alphabet. During SI blocks, participants were asked to continue to go through the alphabet sequence in their head and perform the requested judgement on the letter in their head, while ignoring a distracting random blue letter that was presented on the screen. The specific shape judgement varied across each of three sessions, to reduce the likelihood of participants learning the correct sequence of button presses. Participants judged whether the letters contained either of the following: (i) a curve, (ii) a straight vertical line and (iii) a straight horizontal line. SO and SI blocks alternated and lasted on average 4.5 trials (range, 3–7 trials).

The new task variant used here included the additional factor of distractor type, to explore the effect of distracting socio-affective stimuli on the control and allocation of attention. All trials were pseudo-randomly allocated to one of three distractor conditions: no distractor (50% of the trials), fearful faces (25%) and happy faces (25%). In the latter conditions, the image of either a fearful or happy face was presented centrally behind the letter stimuli. Faces were selected from the NimStim (Tottenham *et al.*, 2009) and NIMH Child Emotional Faces Picture Set (Egger *et al.*, 2011) stimulus sets, from 24 models (12 adult males, 2 adolescent males; 12 adult females, 2 adolescent females), with each model providing both happy and fearful face stimuli. In the no distractor condition, the letter was presented directly on a black background (Figure 1). Faces were 8.1 cm × 6 cm (H × W) in size, and letters measured 2 cm in height (width varied). Participants viewed the screen from ∼45 cm away, giving approximate visual angles of 10.29° (face) and 2.55° (letter). The task was self-paced and, including training and testing phases, lasted on average 9.7 min.

### Statistical analyses

We modelled the effect of *COMT* genotype on Emotional Alphabet task performance using mixed-design repeated measures analysis of variance (ANOVA), with *COMT* genotype and sex as between subject factors, and block type and distractor type as within-subject factors. The first trial in each block (switch trials) was excluded, as performance is known to vary on these trials and the task included too few switch trials to analyse them separately, as in previous studies (Gilbert *et al.*, 2005). Participants were excluded for poor performance if they exhibited either a mean percentage error (PE) rate (*n* = 5) or mean reaction time (RT) for correct trials (*n* = 2) over 3 standard deviations from the overall mean in SI, SO or both block types. Separate ANOVAs were performed for mean PE and mean RT for correct trials. Post hoc *t*-tests are reported with Bonferroni correction.

The ability to maintain and manipulate self-generated information accurately may be closely related to WM, which has previously been found to be associated with *COMT* genotype (Tunbridge *et al.*, 2006; Dickinson and Elvevåg, 2009; Mier *et al.*, 2010; Dumontheil *et al.*, 2011; Witte and Flöel, 2012). Analysis of *COMT* genotype effects on standard measures of WM are reported in a separate paper (Dumontheil *et al.*, 2014). A significant advantage was observed for Met/Met participants on the VSWM and Backwards Digit span tasks. These effects remained significant in the participant sample considered here, which was slightly smaller owing to the exclusions of participants for poor performance on the Emotional Alphabet task (VSWM: *t*(122) = 2.71, *P* = 0.008; Backwards Digit: *t*(99.97) = 2.21, *P* = 0.029). Analyses of genotype effects on the Emotional Alphabet task were therefore also run including measures of WM as covariates in the model, to evaluate the extent to which findings could be accounted for by effects of genetic variation at *COMT* on standard WM performance.

Statistical analysis was carried out in SPSS (version 21), using Greenhouse-Geisser correction when assumptions for sphericity were not met. Analyses were repeated with age included as a covariate to assess whether age differences between the genotype groups accounted for significant genotype effects. There were no main effects of age, nor any significant interactions with age, and all results remained significant. Therefore, we report in the text and plot in relevant figures the estimated standardized means and standard errors obtained from the original repeated measures ANOVAs (see Supplementary Materials for analysis of covariance results).

## RESULTS

### Genetic effects on the Emotional Alphabet task

There was a significant main effect of block type (*F*(1,120) = 37.58, *P* < 0.001, η^2^ = 0.238) on PE. Participants made more errors in SI blocks (Mean = 8.72%, SE = 1.09) than SO blocks (Mean = 2.85%, SE = 0.42). There was no main effect of distractor type (*P* = 0.635) or participant’s sex on PE (*P* = 0.438). There was, however, a significant main effect of *COMT* genotype (*F*(1,120) = 5.59, *P* = 0.020, η^2^ = 0.044), with Val carriers making more errors (Mean = 7.38%, SE = 0.72) than Met/Met participants (Mean = 4.19%, SE = 1.14).

**Fig. 2:**
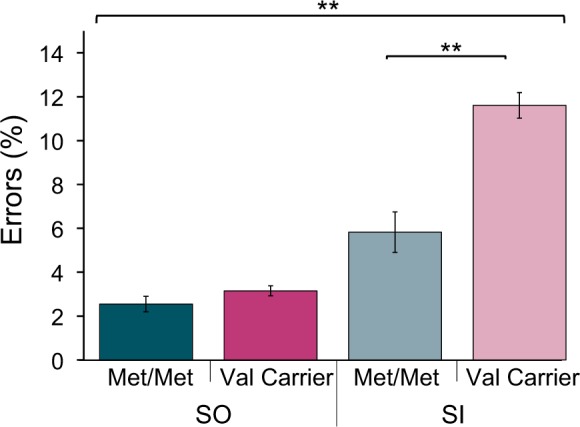
Interaction of block type and *COMT* genotype on mean PE in the Emotional Alphabet task (Mean ± 1 SE). Post hoc independent samples *t*-tests indicated that Val carriers made more errors than Met/Met participants on SI blocks (**P < 0.01).

This main effect of *COMT* genotype was moderated by block type (*F*(1,120) = 7.34, *P* = 0.008, η^2^ = 0.058). Post hoc Bonferroni-corrected independent samples *t*-tests indicated that the interaction was due to a difference between genotype groups on SI blocks (*t*(104.1) = 3.09, *P* = 0.005) and not SO blocks (*P* = 0.871), which suggests the observed main effect of *COMT* genotype was driven by the group difference on SI blocks (Figure 2).

**Fig. 3:**
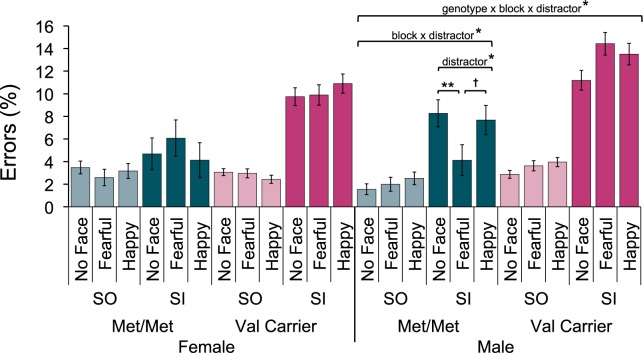
Four-way interaction of block type, distractor type, *COMT* genotype and sex on mean PE in the Emotional Alphabet task (Mean ± 1 SE). Follow-up repeated measures ANOVAs indicate that male Met/Met participants made fewer errors when exposed to a fearful face distractor, specifically in SI blocks (**P < 0.01, *P < 0.05, ^†^*P* < 0.1).

There was also a four-way interaction between block type, distractor type, *COMT* genotype and sex on PE (*F*(1.8, 216.1) = 4.28, *P* = 0.018, η^2^ = 0.034; see Figure 3). To decompose this interaction, the sample was split by sex and separate follow-up ANOVAs (block type x distractor type x *COMT* genotype) were run for male and female participants. For male participants, there was a three-way interaction (*F*(2,116) = 4.39, *P* = 0.015, η^2^ = 0.070), which was not found for female participants (*P* = 0.262). To understand the three-way interaction in males, the male sample was further split by *COMT* genotype and separate ANOVAs were run.

Male Met/Met participants displayed a significant interaction of block type with distractor type (*F*(2,40) = 4.48, *P* = 0.017, η^2^ = 0.183), which was not found in male Val carriers (*P* = 0.281). Follow-up one-way ANOVAs on the Male Met/Met subsample demonstrated that an effect of distractor type was only found in SI blocks (*F*(2,40) = 5.16, *P* = 0.010, η^2^ = 0.205; SO blocks: *P* = 0.490). Post hoc Bonferroni-corrected pairwise comparisons indicated that in SI blocks, male Met/Met participants made fewer errors on fearful face trials compared with other trial types (fearful *vs* no distractor, *P* = 0.002; fearful *vs* happy face, *P* = 0.085; no distractor *vs* happy face, *P* = 1.00). To summarize this four-way interaction, male, but not female, participants showed a sensitivity to the affective distractors that depended on *COMT* genotype. Male Met/Met participants displayed a specific improvement in accuracy in SI trials when a fearful face distractor was presented.

There was a significant main effect of block type on mean RT (*F*(1,120) = 192.83, *P* < 0.001, η^2^ = 0.616): participants were slower in SI blocks (Mean = 1206 ms, SE = 27) than SO blocks (Mean = 993 ms, SE = 22). However, there were no genetic effects on RT (*P* values > 0.140), nor any other main effects or interactions (*P* values > 0.071).

### Role of WM in genetic effects

Analyses of significant *COMT* genotype effects were also run including measures of standard WM as covariates in the model. Including Backward Digit span as a covariate did not alter the significance of the genetic effects and genetic interaction effects reported above. However, when VSWM score was included as a covariate, there was no longer a significant main effect of *COMT* genotype (*P* = 0.125), while the significant interaction between block type and *COMT* genotype became trend level (*P* = 0.064). The four-way interaction between block type, distractor type, *COMT* genotype and sex remained significant (*P* = 0.017).

## DISCUSSION

We investigated the effect of the Val^158^Met polymorphism, a common single nucleotide polymorphism of the *COMT* gene, on the flexible modulation of the balance between processing self-generated and perceptually derived information and the ability to attend to and manipulate self-generated information. We also examined the influence of socio-affective distractors on task performance. To do so, we designed an emotional variant of the Alphabet task (Gilbert *et al.*, 2005), which required participants to flexibly select perceptually derived or self-generated information, in the presence or absence of socio-affective perceptual distractors. Our aim was to investigate the role of prefrontal dopamine transmission on these cognitive processes, using genetic variation and associated individual differences as tools to study indirectly the role of neurotransmitter systems on behaviour.

### Variation at *COMT* and the ability to attend toward and manipulate self-generated information

We hypothesized that individuals homozygous for the Met allele would show superior performance on SI blocks of the Emotional Alphabet task, due to increased prefrontal dopamine availability. This is thought to result in more efficient functioning of the PFC, which supports the selection and manipulation of SI (relative to SO) information (Gilbert *et al.*, 2005). We found a main effect of *COMT* genotype and an interaction between *COMT* genotype and block type on task performance. Both effects were driven by a greater number of errors made by Val carriers compared with Met/Met participants in SI blocks, in which participants were required to ignore perceptually derived (SO) information and instead process self-generated (SI) information. This suggests Val carriers had more difficulty in continuing through the alphabet sequence in their head and manipulating the relevant self-generated letter representations than Met/Met individuals did. Alternative, yet not mutually exclusive, explanations for this difficulty are that Val carriers had more difficulty suppressing the processing of the irrelevant letter stimuli presented during SI blocks, or in keeping active the current task goals in memory. Such an explanation would be consistent with a recent study that found Val carriers show a larger sensitivity to interference on a modified Stroop task than Met homozygotes (Jaspar *et al.*, 2014). Our finding and Jaspar *et al.*'s (2014) results could be interpreted within the framework of the dual mechanisms theory of control (Braver *et al.*, 2007; Braver, 2012), suggesting that the lower dopamine levels in Val carriers result in less efficient sustained (‘proactive’) cognitive control. There were no effects of *COMT* genotype on RTs, consistent with previous associations of *COMT* genotype with executive function, which predominately relate to measures of accuracy (Tunbridge *et al.*, 2006; Dickinson and Elvevåg, 2009; Witte and Flöel, 2012; Jaspar *et al.*, 2014). This suggests that the difference between Val carriers and Met homozygotes was in the ability to maintain and manipulate self-generated information accurately over a sustained block of trials, rather than in the speed of processing self-generated thoughts. This ability to maintain and manipulate self-generated information may be closely related to WM, which has previously been found to be associated with *COMT* genotype (Tunbridge *et al.*, 2006; Dickinson and Elvevåg, 2009; Dumontheil *et al.*, 2011; Witte and Flöel, 2012).

To evaluate the extent to which effects of *COMT* genotype on the Emotional Alphabet task could be accounted for by genetic variation in standard WM performance, analyses were repeated while controlling for performance on standard measures of visuospatial and verbal WM. When VSWM score was included as a covariate in the model, there was no longer a main effect of *COMT* genotype, or a significant interaction between block type and *COMT* genotype. This suggests, at least to some extent, that the influence of prefrontal dopamine levels on the ability to attend to and manipulate SI information may operate via the same mechanism by which prefrontal dopamine influences VSWM abilities. The existence of shared genetic variance on performance on these tasks is consistent with the conceptualization of WM as a sub-component of executive function (Dickinson and Elvevåg, 2009). While Backwards Digit span score accounted for some of the genetic variance, this effect was not as pronounced as that of VSWM. This may suggest a greater overlap between cognitive processes required in VSWM and the Emotional Alphabet task, than between Backwards Digit span and the Emotional Alphabet task, or that the VSWM task we used was more sensitive to effects of *COMT* genetic variation. The shape judgment required of participants in the Emotional Alphabet task may have more greatly loaded their VSWM capacity than the need to remember a single letter of the alphabet loaded their verbal WM capacity.

Effects of *COMT* genotype on executive function have not always been replicated (Barnett *et al.*, 2008; Dickinson and Elvevåg, 2009; Witte and Flöel, 2012), and some authors have suggested that effects are dependent on the specific task being studied, and the specific cognitive demands that underlie it (Cools and D’Esposito, 2011). It has been argued that in healthy populations, neuropsychological tasks used to measure executive function and WM may show limited variance (Dickinson and Elvevåg, 2009). Our results demonstrate that accuracy on the Emotional Alphabet task was sensitive to variation in *COMT* genotype, validating this task for future use.

### Sex effects and *COMT* variation on the influence of affective distractors

A four-way interaction was found between block type, distractor type, *COMT* genotype and sex. Follow-up analyses showed that male, but not female, participants showed a genotype-dependent sensitivity to the presence of socio-affective distractors. For male participants, those homozygous for the Met allele displayed a specific improvement in accuracy in SI trials when a fearful face distractor was presented. This effect was not accounted for by the effect of genetic variation on WM, which may suggest that the shared genetic variance on tasks of executive function and WM is different to genetic effects on socio-affective cognition and emotional regulation. We did not have specific predictions regarding the direction of the effects of socio-affective distractors on task performance, as there are multiple theories pertaining to the precise nature of attentional biases towards emotionally salient stimuli within the emotion-related psychopathology literature (Bar-Haim *et al.*, 2007; Ladouceur *et al.*, 2009). One theory is that there is a tendency to disengage attentional resources from negative stimuli, but not positive stimuli (Williams *et al.*, 1997). This might offer an explanation as to why accuracy was increased in the presence of a fearful face in SI trials: male Met/Met participants may have disengaged from the presented perceptual stimuli (both the fearful face and the superimposed irrelevant letter), thus improving their ability to attend to and process self-generated information.

There are a number of limitations to the current study. Dividing the genotype groups by sex resulted in small sub-group sizes, and therefore, these results remain exploratory, and only tentative conclusions can be made. However, the fact that affective distractors showed genotype effects in only male participants is consistent with previous research suggesting that sex may moderate the effects of COMT on prefrontal cognition (Harrison and Tunbridge, 2008). Oestrogen has a regulatory effect on *COMT* expression, and in females variation in oestrogen levels has been shown to modulate prefrontal activity during a WM task (Jacobs and D’Esposito, 2011), within *COMT* genotype groups. While speculative, it is possible that in a female population there is a greater variance in dopamine levels within each genotype group, and therefore, genotype effects are more difficult to detect at a group level.

We acknowledge that the distribution of participants’ sex was not well matched, and participants’ age differed significantly between *COMT* groups (see Supplementary Materials for further details on group matching). These factors can moderate the effects of *COMT* genotype on cognition (Barnett *et al.*, 2007; Harrison and Tunbridge, 2008; Dumontheil *et al.*, 2011); therefore, we attempted to minimize their influence. Sex was included in all analyses, and all analyses were repeated with age entered as a covariate, which did not affect the results. It should also be noted that the genotype groups did not differ on self-reported measures of anxiety (Spielberger *et al.*, 1983). Our sample consisted of psychiatrically healthy participants and it is conceivable that genotype effects on affective processing are more likely to be detected at the level of variations in cognitive processing, rather than in terms of overt anxiety symptomatology (Meyer-Lindenberg and Weinberger, 2006).

## CONCLUSION

This study provides evidence for a role of genetic variation on the ability to select and manipulate self-generated thoughts, an aspect of executive function that has not previously been studied in relation to dopaminergic function. This genetic variance appears to be to some extent overlapping with genetic variation in WM, suggesting that both processes are affected by prefrontal dopamine levels. We also find preliminary evidence of sexually dimorphic effects of *COMT* genotype on the influence of socio-affective distractors on executive function, suggesting that the interplay between the prefrontal dopaminergic system and socio-affective processing regions may be sensitive to sex differences. Increasing our knowledge of the role of dopamine in cognitive processes implicated in affective disorders, including executive function, attention and socio-affective processing, is critical in understanding how individual differences may confer risk for such disorders, and the mechanisms underlying such risk.

## SUPPLEMENTARY DATA

Supplementary data are available at *SCAN* online.

### Conflict of Interest

None declared.

Supplementary Data
