# Supplementary material for: Influence of COMT genotype and affective distractors on the processing of self-generated thought
Source: Soc Cogn Affect Neurosci. 2014 Sep 3;10(6):777–82. doi: 10.1093/scan/nsu118 (PMC4305337; doi:10.1093/scan/nsu118)
Supplement: Supplementary Data [file supp_10_6_777__index.html]

Influence of COMT genotype and affective distractors on the processing of self-generated thought — Influence of COMT genotype and affective distractors on the processing of self-generated thought — Supplementary Data 

# Influence of *COMT* genotype and affective distractors on the processing of self-generated thought

## Supplementary Data

files

**Files in this Data Supplement:**

- Supplementary Data - doc file
