# Supplementary material for: Influence of COMT genotype and affective distractors on the processing of self-generated thought
Source: Soc Cogn Affect Neurosci. 2014 Sep 3;10(6):777–82. doi: 10.1093/scan/nsu118 (PMC4305337; doi:10.1093/scan/nsu118)
Supplement: Supplementary Data [file supp_nsu118_scan-14-185-File006.doc]

**Supplementary Materials**

**Genetic Analysis**

**Genetic sampling and analysis**. Saliva samples were collected using OG-500, Oragene-DNA self-Collection Kit, Oragene.DNA, as per protocol suggested by Oragene.DNA.^7^ DNA was extracted from saliva samples at the Department of Molecular Neuroscience at the Institute of Neurology, UCL. DNA was extracted using the OG-L2P DNA extraction kit (DNA Genotek Inc., Ontario, Canada) as per protocol suggested by Oragene.DNA (http://www.dnagenotek.com/US/pdf/PD-PR-006.pdf).

The analysis of the single nucleotide polymorphism (SNP) was carried out by AROS, University of Aarhus, Denmark. The *COMT* rs4680 SNP in exon 4 of the gene characterised by an A/G substitution, which causes the Val^158^Met polymorphism. The SNP was determined using the TaqMan-based genotyping technology from Applied Biosystems (Foster City, California). Reactions and analysis were performed in a 384-well plate format. All samples were normalized to 5ng/µl of DNA. The reaction components for each genotyping reaction were as follows: 2.5 µl TaqMan master mix, 0.25 µl TaqMan assay X20, 1.25 µl water resulting in a total volume of 4.0 µl + 1 µl template genomic DNA with a concentration of 5ng/µl. The reaction was analysed using an Applied Biosystems 7900 Fast RT-PCR instrument. Included in the analysis were three negative controls (no template control) and five positive controls (known DNA samples and known SNP assays).

**Data quality control.** The genotyping was validated using a set of five control samples with genotype data available through the Coriell Institute for Medical Research. There was a 100% concordance with the data from Coriell Institute for Medical Research. The genotyping was unsuccessful for eight of 161 (5%) participants due to poor quality of the sample.

**Allele frequency distributions.** We observed an allele frequency distribution of .52 Met and .48 Val, which is comparable to previously reported allele frequency distributions of .48 Met and .52 Val (Hapmap European sample). The Hardy-Weinberg equilibrium test indicated that the genotypic frequency distribution was in equilibrium on the *COMT* polymorphism (*X*^2^=.78, *p*=.378). We identified 36 subjects (29%) with the Met/Met genotype, 57 with Met/Val (46%) and 31 with Val/Val (25%).

**Participants**

**Matching of genotypic groups.** Pearson’s chi-squared tests indicated that the two *COMT* genotype groups (Met/Met, Val carriers) did not differ significantly in sex (*X*^2^(1)=2.01, *p*=.156), or ethnicity: Caucasian (White British (*n*=52), White European (*n*=32)) versus non-Caucasian (Black African and Caribbean (*n*=12), Indian, Pakistani and Bangladeshi (*n*=19), Mixed Ethnic background (*n*=8); *X*^2^(1)=2.00, *p*=.157). Independent samples t-tests indicated that the *COMT* groups were well-matched on state anxiety (*t*(79.58)= .276, *p*=.783), trait anxiety (*t*(121)= .986, *p*=.326; note that trait anxiety was missing for one participant) and verbal IQ *(t*(120)= .336, *p*=.737; note that IQ data were missing for two participants). However, age differed significantly between the groups (*t*(122)=2.01, *p*=.047), Met/Met participants being slightly younger (Mean=25.25, SD=3.20) than Val carriers (Mean=26.74, SD=3.95).

**Matching of sex groups.** Pearson’s chi-squared test indicated that male and female groups did not differ significantly in ethnicity (coded as Caucasian versus non-Caucasian (includes participants of mixed Caucasians and non-Caucasians ethnicity)) (*X*^2^(1)=1.24, *p*=.267). Independent samples t-tests revealed that male and female groups did not significantly differ in state anxiety (*t*(122)=.390, *p*=.697), trait anxiety (*t*(121)=.321, *p*=.749), verbal IQ (*t*(120)= .684, *p*=.495) or age (*t*(112.4)=1.575*, p*=.118).

**Analyses Including Age as a Covariate** All analyses were repeated with age included as a covariate to assess whether age differences between the genotype groups accounted for significant genotype effects. All task factors, genetic factors and interactions remained significant and are presented below, along with *post-hoc* tests that make comparisons between the two genotype groups. Similarly, including age as an additional covariate in the analyses covarying for WM did not alter the pattern of results.

**Genetic effects on the Emotional Alphabet task.** There was a significant main effect of block type (*F*(1,119)=37.34, *p*<.001, *η*^2^ =.239) on PE, with participants making more errors in SI blocks than SO blocks There was no main effect of distractor type (*p*=.614) or participant’s sex on PE (*p*=.474). There was a significant main effect of *COMT* genotype (*F*(1,119)=4.95, *p*=.028, *η^2^*=.040), with Val carriers making more errors than Met/Met participants.

This main effect of *COMT* genotype was moderated by block type (*F*(1,119)=7.34, *p*=.011, *η^2^*=.054). *Post-hoc* Bonferroni-corrected one-way ANCOVAs indicated a difference between genotype groups on SI blocks (*F*(1,121)=5.68, *p*=.038) and not SO blocks (*p*=.100).

There was a four-way interaction between block type, distractor type, *COMT* genotype and sex on PE (*F*(1.8,214.1) =4.21, *p*=.019, *η^2^*=.034). Separate follow-up ANOVAs (block type x distractor type x *COMT* genotype) were run for male and female participants. For male participants there was a three-way interaction (*F*(2,114)=3.794, *p*=.025, *η^2^*=.062), which was not found for female participants (*p*=.296). There was a significant main effect of block type on mean RT (*F*(1,119)=190.53, *p*< .001, *η^2^*=.616): participants were slower in SI blocks than SO blocks . There were no genetic effects on RT (*p*s>.179), nor any other main effects or interactions (*p*s>.096).

**Role of working memory in genetic effects.** Including both age and Backward Digit span as covariates did not alter the significance of the genetic effects and genetic interaction effects reported above, similar to the results found without the age covariate (see main text). When age and VSWM score were included as covariates, there was no longer a significant main effect of *COMT* genotype (*p*=.153), while the significant interaction between block type and *COMT* genotype became trend level (*p*=.076). The four-way interaction between *COMT* genotype, sex, block type and distractor type remained significant (*p*=.018).

**Supplementary References**

Hapmap European sample. http://www.ncbi.nlm.nih.gov/SNP/snp_ref.cgi?rs=4680/ (accessed 12 Nov 2013).
